# A PITX2–HTR1B pathway regulates the asymmetric development of female gonads in chickens

**DOI:** 10.1093/pnasnexus/pgad202

**Published:** 2023-06-19

**Authors:** Zhelun Peng, Qiu Man, Lu Meng, Sheng Wang, Hao Cai, Chuansheng Zhang, Xianyao Li, Heng Wang, Guiyu Zhu

**Affiliations:** College of Animal Science and Technology, Shandong Provincial Key Laboratory of Animal Biotechnology and Disease Control and Prevention, Shandong Agricultural University, Taian 271018, China; College of Animal Science and Technology, Huazhong Agricultural University, Wuhan 430070, China; College of Animal Science and Technology, Shandong Provincial Key Laboratory of Animal Biotechnology and Disease Control and Prevention, Shandong Agricultural University, Taian 271018, China; College of Animal Science and Technology, Huazhong Agricultural University, Wuhan 430070, China; College of Animal Science and Technology, Shandong Provincial Key Laboratory of Animal Biotechnology and Disease Control and Prevention, Shandong Agricultural University, Taian 271018, China; College of Animal Science and Technology, Huazhong Agricultural University, Wuhan 430070, China; College of Animal Science and Technology, Shandong Provincial Key Laboratory of Animal Biotechnology and Disease Control and Prevention, Shandong Agricultural University, Taian 271018, China; College of Animal Science and Technology, Shandong Provincial Key Laboratory of Animal Biotechnology and Disease Control and Prevention, Shandong Agricultural University, Taian 271018, China; College of Animal Science and Technology, Hebei Normal University of Science and Technology, Qinhuangdao 066600, China; College of Animal Science and Technology, Shandong Provincial Key Laboratory of Animal Biotechnology and Disease Control and Prevention, Shandong Agricultural University, Taian 271018, China; College of Animal Science and Technology, Shandong Provincial Key Laboratory of Animal Biotechnology and Disease Control and Prevention, Shandong Agricultural University, Taian 271018, China; College of Animal Science and Technology, Shandong Provincial Key Laboratory of Animal Biotechnology and Disease Control and Prevention, Shandong Agricultural University, Taian 271018, China; College of Animal Science and Technology, Huazhong Agricultural University, Wuhan 430070, China

**Keywords:** PITX2, chicken gonad, asymmetrical development, HTR1B, ovary

## Abstract

All female vertebrates develop a pair of ovaries except for birds, in which only the left gonad develops into an ovary, whereas the right gonad regresses. Previous studies found that the transcription factor Paired-Like Homeodomain 2 (PITX2), a key mediator for left/right morphogenesis in vertebrates, was also implicated in asymmetric gonadal development in chickens. In this study, we systematically screened and validated the signaling pathways that could be targeted by Pitx2 to control unilateral gonad development. Integrated chromatin immunoprecipitation sequencing (ChIP-seq) and RNA sequencing (RNA-seq) analyses indicated that Pitx2 directly binds to the promoters of genes encoding neurotransmitter receptors and leads to left-biased expression of both serotonin and dopamine receptors. Forcibly activating serotonin receptor 5-Hydroxytryptamine Receptor 1B (HTR1B) signaling could induce ovarian gene expression and cell proliferation to partially rescue the degeneration of the right gonad. In contrast, inhibiting serotonin signaling could block the development of the left gonad. These findings reveal a PITX2–HTR1B genetic pathway that guides the left-specific ovarian growth in chickens. We also provided new evidence showing neurotransmitters stimulate the growth of nonneuronal cells during the early development of reproductive organs well before innervation.

**Significance Statement**

In most animals, the two gonads develop symmetrically to make the pair of testes or ovaries. However, birds develop only a left ovary. A possible role of Pitx2 in this asymmetric ovarian development has been proposed but not validated. We discovered that Pitx2 directly induces expression of specific neurotransmitter receptors, leading to left-biased activation of the serotonin receptor. This activation stimulates cell proliferation and ovarian gene expression, contributing to the development of the left ovary. The induction of the serotonin pathway in the right gonad could partially rescue the development of the regressing gonad. We provide the first evidence that neurotransmitters may regulate the left/right asymmetric development of avian ovaries, occurring before the formation of peripheral neurons in the tissue.

## Introduction

Birds exhibit an unusual asymmetry in terms of the development of gonads. The female left gonad generates a functional ovary, whereas the right gonad regresses. In males, both left and right gonads would develop into testes, although a subtle left/right difference was also identified. Around 3.5 days in chicken embryos (E3.5), the gonads arise on the ventromedial surface of each mesonephros (1). As sex-specific differentiation proceeds around E4.5, the gene expression and epigenetic modifications already show dramatic differences between male and female gonads, although they are morphologically identical at this stage (2). In females, only the left gonads develop normally and finally form functional ovaries, while the right ones gradually degenerate and finally disappear. From E6.5 onwards, the left gonads were growing and appearing bigger in size than the right one and the cortex of the left gonad is thicker than that in the right (1). Several reports studied this female-specific left/right asymmetrical development of gonads by mainly focusing on gene transcription (3, 4) and chromatin activation (5). A couple of genes and related signaling pathways were distinctively expressed between left and right gonads. For example, estrogen receptor α (ERα) was predominantly expressed in the cortex of the left but not the right gonad (6) and BMP7 was left/right asymmetrically produced at the beginning of gonad formation (7). Nevertheless, whether these genes could directly establish the left-specific development of female gonads remains unclear.

Paired-Like Homeodomain 2 (Pitx2), a multifunctional paired-like homeodomain transcription factor, is an important player in the establishment of the left/right axis in vertebrates. Pitx2 is specifically expressed in the left lateral mesoderm of mice and zebrafish, which can guide the development and placement of multiple internal organs such as the heart and gut (8–10). Accordingly, the deletion or ectopic expression of Pitx2 can lead to organ-specific left/right lateralization defects in many animal models (11, 12). As to the reproductive organs, Pitx2 was expressed in both sides of the developing gonads of female rats (13), but currently, there is no evidence to suggest that Pitx2 could regulate the bilateral development of mammalian gonads. In contrast, chicken Pitx2 was only expressed in the cortex of the female left gonad beginning from the formation of gonadal primordia up to E12. Ectopic expression of Pitx2 in the right regressing gonad can stimulate the formation of cortex-like structures and induce the meiotic entry of germ cells (14, 15). Furthermore, Pitx2 can positively regulate ERα, CCND1, CCNA1, LEF1, and SRSF1 to promote the proliferation of cells in the left cortex, eventually leading to the growth of the left gonad but not the right one (16–19). Nevertheless, the specific factors that drive the left-specific expression of Pitx2 in chicken gonads were unknown. In addition, as a transcription factor, the chromatin binding patterns and direct downstream target genes of Pitx2 in the chicken gonad remain to be discovered.

In this study, we analyzed both the upstream signaling pathways and downstream targets of Pitx2 in female chicken gonads. We identified the proximal regulatory regions with the strongest promoter activity of chicken Pitx2 and the transcription factors that could contribute to the situs-specific expression of Pitx2 between the pair of gonads. We also profiled the whole-genome binding sites of chicken Pitx2 and identified the potential target factors that may lead to the asymmetric development of female gonads. Specifically, we provided the first line of evidence to suggest the role of Pitx2-induced neurotransmitter receptors in promoting gonadal cell proliferation and ovarian development. The current study provided new mechanisms for explaining the left/right asymmetric development of female ovaries in avian species.

## Results

### Gene expression analysis revealed the asymmetric expression of Pitx2 between left and right gonads

**Fig. 1.**
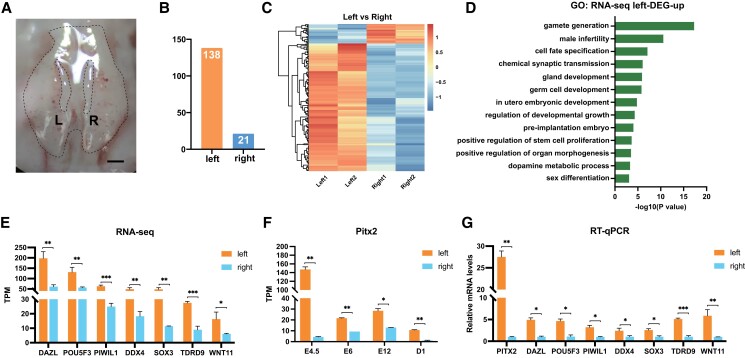
Expression analysis of the situs-specific genes. A) The morphology of female gonads at E4.5. Scale bar: 1 mm. B) The number of DEGs between left and right gonads at E4.5. C) Heatmap of DEGs between left and right gonads at E4.5 (two duplicates). D) GO analysis of up-regulated DEGs in the left. E) The RNA expression of representative DEGs. F) PITX2 expression was consistently higher in the left than the right during gonad development. G) RT-qPCR results of PITX2 and germ cell–related genes at E4.5. Data are mean ± SEM; *P < 0.05, **P < 0.01, ***P < 0.001 after the *t* test; *n* = 4 in each group.

We first analyzed the overall transcriptome of the gonad and identified the differentially expressed genes (DEGs) between female left and right gonads at E4.5. At this time point, the pair of the two gonads looked almost identical in morphology (Fig. 1A) but already showed very different gene expression patterns. RNA sequencing (RNA-seq) was performed in duplicate, and each biological replicate included at least five gonads from each left or right side. In general, transcriptional activity is higher in the left than in the right. A total of 138 DEGs were left-biased, and 21 DEGs were right-biased (Fig. 1B and C). Gene Ontology (GO) analysis revealed that the left-biased genes were enriched in biological processes related to gonadal development such as “gamete generation,” “cell fate specification,” “gland development,” and “germ cell development” (Fig. 1D). Moreover, signaling pathways related to neurotransmitter transmission and metabolism such as “chemical synaptic transmission” and “dopamine metabolic process” were also enriched in the left (Fig. 1D). Next, we employed RT-qPCR to validate the situs-biased expression patterns of the representative genes involved in female gonad and germ cell development, such as DAZL (20), Pou5f3 (21), PIWIL1 (22), DDX4 (23), SOX3 (24), TDRD9 (3), and WNT11 (25). The RT-qPCR results confirmed that RNA-seq is reliable (Fig. 1E and G).

As expected, Pitx2 expression was left-biased. Combined with the available RNA-seq data of previous studies from E6–E12 gonads and D1 ovaries, we observed that Pitx2 expression levels were dramatically higher in the left than the right throughout gonad development (Fig. 1F and G). It indicates that Pitx2 could play an important role during the growth of left gonads into ovaries. On the other hand, the 21 right-biased genes including genes such as LAPTM4B (26) and RICTOR (27) are key genes involved in cell autophagy that may contribute to tissue degradation (Fig. S1), which further confirmed the tissue phenotype of the right-sided degeneration in chicken embryos.

### Identification of the *cis*- and *trans*-regulatory elements of chicken Pitx2 in chicken gonads

**Fig. 2.**
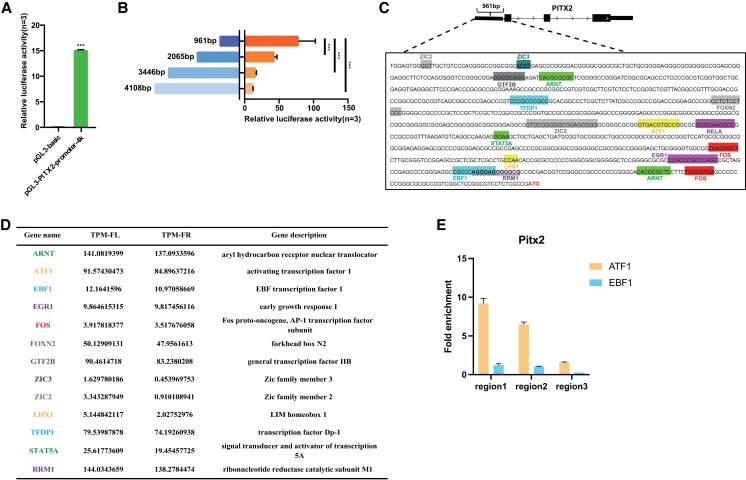
Identification of the potential transcription factors that contribute to situs-specific Pitx2 expression. A) Luciferase reporter assay of the PITX2-4k promoter. B) The activity of different truncated promoter regions as shown by the luciferase signal. Note that the 1k length promoter is the most active region. C) Prediction of transcription factors could bind to the most active regions of the promoter. The start codon is in labelled at the end of sequence. D) The mRNA expression of predicted transcription factors between the pair of gonads. E) The ChIP-qRCR results showed that the pitx2 promoter fragments were significantly enriched in the ATF1 and EBF1 ChIP pull-down DNA compared with the input DNA without antibody enrichment. Fold enrichment presents ATF1 and EBF1 ChIP results relative to the negative sample at the three regions around the pitx2 promoter. Data are mean ± SEM; ***P < 0.001 after the *t* test; *n* = 5 in each group.

To understand the transcriptional regulation of the Pitx2 gene in chicken embryonic gonads, we first investigated the core promoter region at the proximal end of the gene locus. We cloned the 4 kb genomic sequence upstream of the translation initiation site into the luciferase reporter (Fig. S2) and examined luciferase activity in embryonic fibroblast cells. We found that the promoter activity of the pGL3-PITX2-promoter-4k vector was significantly higher than that of the pGL3-Basic control vector (Fig. 2A), confirming the active state of the promoter of the chicken Pitx2 gene. Subsequently, we generated a series of 5′ truncated luciferase reporter constructs based on the −4,108 bp sequence and designated them as pGL3-PITX2-promoter-1k (−961 bp), pGL3-PITX2-promoter-2k (−2,065 bp), and pGL3-PITX2-promoter-3k (−3,446 bp) (Fig. S2). Luciferase activity derived from differently sized fragments of the Pitx2 promoter was examined, and pGL3-PITX2-promoter-1k showed significantly higher activity than that of other vectors after transfection of embryonic fibroblast cells (Fig. 2B), indicating the most proximal 961 bp region could play a major role in Pitx2 transcriptional regulation. We further analyzed the sequence of this core promoter by predicting the potential transcription factor binding motifs located within this most active region (Fig. 2C). By combining the putative transcription factors and the genes whose expression is higher in the left than the right from the gonadal transcriptome, the potential upstream regulatory factors of Pitx2 in chicken gonads are determined. Among these transcription factors, some are closely associated with biological processes involved in cell proliferation, transcriptional activation, and gonadal development such as LHX1 (28), ATF1 (29), STAT5A (30), and other genes (Fig. 2D). Furthermore, we selected candidate upstream transcription factors and performed chromatin immunoprecipitation (ChIP)-qPCR assay in gonadal cells to validate the interaction between the transcription factors and the Pitx2 regulatory sequences. ChIP-qPCR showed that the Pitx2 promoter sequences were significantly enriched in the ATF1 and EBF1 immunoprecipitated DNA compared with the input control (no antibody), indicating that ATF1 and EBF1 indeed bind to the promoters of Pitx2 in chicken gonads (Fig. 2E). Together, the left/right asymmetric distribution of these transcription factors in the female chicken gonads could drive the left-specific expression of Pitx2.

### The identification of Pitx2 binding sites in the chicken genome by ChIP sequencing

**Fig. 3.**
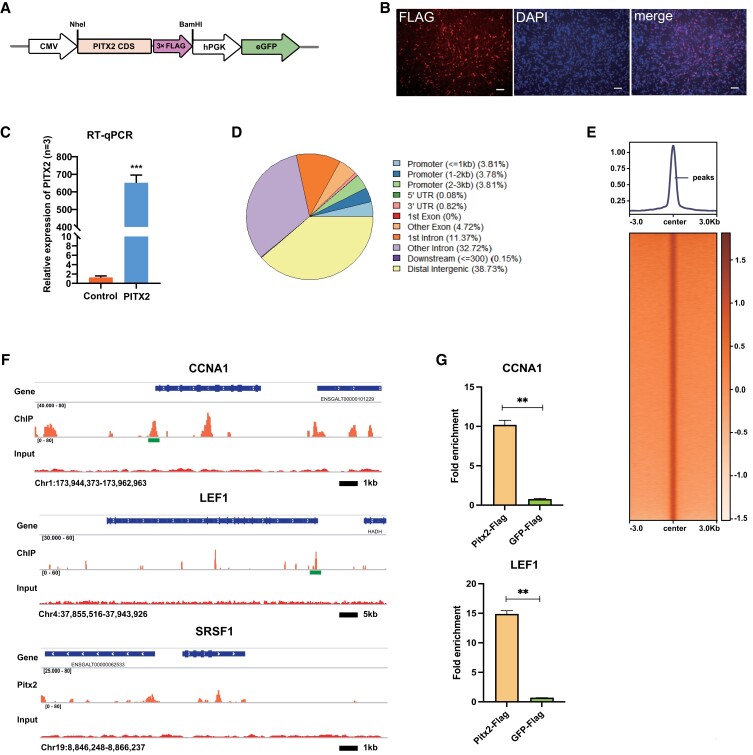
Basic features and annotations of PITX2 chromatin binding sites in chicken cells. A) Schematic diagram of PITX2-FLAG fusion expression plasmid. B) Detection of the PITX2-FLAG fusion protein (left) and Pitx2 mRNA (right) in chicken cells. Scale bars: 100 μm. C) Detection of Pitx2 mRNA by RT-qPCR. D) The distribution of Pitx2 peaks across the chicken genome. E) The enrichment of Pitx2 peaks. F) Integrative Genomics Viewer (IGV) tracks show the Pitx2 binding peaks on target genes, such as CCNA1, LEF1, and SRSF1 loci. IGV examples of Pitx2 binding profiles of the selected target genes (CCNA1, LEF1, and SRSF1). The ChIP track represents the peak signal after subtracting the nonspecific input. The green block regions were used for ChIP-qPCR validation experiments. G) ChIP-qPCR of the Pitx2 downstream target genes (CCNA1 and LEF1) in the FLAG antibody immunoprecipitated DNA from the Pitx2-Flag and GFP-Flag (control) cells. Data are mean ± SEM; **P < 0.01 after the *t* test; *n* = 4 in each group.

Next, we continued to investigate the downstream targets of Pitx2 in chicken gonads. Because no suitable Pitx2 antibody was available in chickens and due to the lack of cross-species reactivity of Pitx2 antibodies from other species, we constructed a PITX2-FLAG in-frame fusion expression vector to enable the ChIP assay based on the affinity FLAG antibody (Fig. 3A). After transfecting the overexpression vector into chicken embryonic fibroblasts, FLAG immunofluorescence staining showed that the proportion of cells expressing Pitx2 was >50% (Fig. 3B). RT-qPCR also showed that Pitx2 mRNA was significantly increased (Fig. 3C). We also tried the primary gonadal cell transfection followed by ChIP, but the ChIP efficiency was too low to proceed with further analysis. Nevertheless, ChIP experiments were performed in fibroblasts transfected with the Pitx2 overexpression vector using the FLAG antibody and two biological replicates were sequenced. Information and mapping summary of ChIP sequencing (ChIP-seq) data are listed in Supplementary Table S2. Analysis of the sequencing results showed that the correlation coefficient between the two sample repeats was 0.83, which confirmed the high repeatability (Fig. S4). Next, the two bam files were combined for subsequent peak calling analysis. A total of 32,728 Pitx2 peaks were identified, and the binding enrichment across the chicken genome was highly specific (Fig. 3E). The peak position annotation showed that ∼22% of the peaks were located on the proximal regulatory regions (promoter and first intron) of the gene, and >38% of the peaks were located in the distal intergenic regions (Fig. 3D). We also identified that the classical Pitx2 target genes, such as CCNA1, LEF1, and SRSF1, were specifically bound by Pitx2 at their respective regulatory regions, validating the accuracy of the ChIP-seq assay (Fig. 3F). The ChIP-qPCR assay also confirmed the interaction of Pitx2 protein with the regulatory sequences of CCNA1 and LEF1 (Figs. 3G and S3). These data suggest that Pitx2 could regulate target gene expression by binding to both proximal and distal regions.

### Neurotransmitter receptor HTR1B and DRD4 genes are directly targeted by Pitx2

**Fig. 4.**
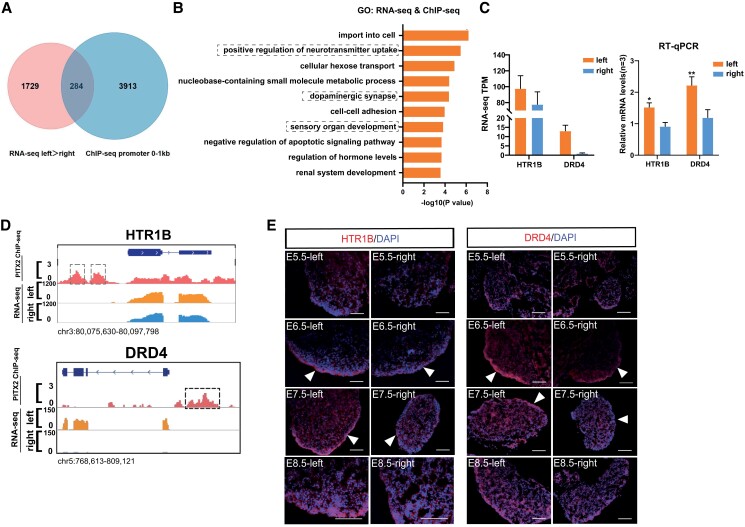
Identification of HTR1B and DRD4 as downstream target genes of PITX2. A) Combined overlap analysis of genes with left gonad-specific expression in RNA-seq and genes with Pitx2 peaks located within 0–1 kb of TSS in ChIP-seq. B) GO analysis of overlapping genes. C) The expression of HTR1B and DRD4 was confirmed by RNA-seq and RT-qPCR. D) IGV tracks show the Pitx2 binding peaks on HTR1B and DRD4 loci. E) Immunofluorescence staining results of HTR1B and DRD4 in female gonads during development. Note the much stronger HTR1B and DRD4 signals in the left than the right gonad, particularly in the cortex regions (arrowheads). Scale bars: 100 μm. Data are mean ± SEM; *P < 0.05, **P < 0.01, ***P < 0.001.

To identify the target genes of Pitx2, ChIPseeker was employed to obtain a preliminary list of genes associated with Pitx2 binding sites. We obtained 4,197 genes that have Pitx2 peaks located within 0–1 kb of the promoters, and these genes could be directly targeted by Pitx2. Next, we overlapped these potential Pitx2 target genes with the genes that exhibit higher expression in the left than in the right gonad in the transcriptome data and obtained 284 candidate target genes of Pitx2 in the gonad (Fig. 4A). These 284 genes could be directly activated by Pitx2 via binding at the promoter region and also show left-biased expression patterns similar to Pitx2 in chicken gonads. We considered these genes would be the key Pitx2 downstream genes that can stimulate the development of the left but not the right gonad. GO analysis showed that these genes were highly enriched in functions related to neurotransmitter and organ development, such as “positive regulation of neurotransmitter uptake,” “dopaminergic synapses,” “hormone level regulation,” and “sensory organ development” (Fig. 4B). In addition, KEGG analysis of these 284 genes also suggests that the “dopaminergic synapse” and “serotonergic synapse” pathways were most enriched (Fig. S5). Hence, we hypothesized that certain neurotransmitter-related pathways and genes may be regulated by Pitx2 to promote the development of the left gonad.

We selected representative neurotransmitter receptor genes that were targeted by Pitx2 for further functional validation experiments. 5-Hydroxytryptamine Receptor 1B (HTR1B) is a receptor of 5-hydroxytryptamine (5-HT), which can interact with serotonin to regulate the function of the central system and is an integral part of the monoaminergic system (31). Dopamine Receptor D4 (DRD4) is a membrane receptor for dopamine and belongs to the G protein–coupled receptor family, which can activate downstream signaling pathways after binding to dopamine (32). We confirmed that the mRNA expression of HTR1B and DRD4 was left-biased in the gonads with RT-qPCR (Fig. 4C). In addition, Integrative Genomics Viewer (IGV) showed the specific Pitx2 binding peaks on the promoter regions of these two genes, and consequently, their expression levels were higher in the left than the right (Fig. 4D). Next, immunofluorescence staining was performed in the sections of both left and right gonads from E5.5–E8.5 female embryos to detect the expression of HTR1B and DRD4 proteins. The results showed that both HTR1B and DRD4 are indeed expressed higher in the left than the right during chicken gonad development. Furthermore, these two neurotransmitter receptor proteins are preferentially distributed in the cortex of gonads (Fig. 4E). Thus, we confirmed that the neurotransmitter-related signaling pathways may play an important role in the asymmetric development of female gonads in chickens.

### HTR1B is regulated by Pitx2 and promotes the proliferation of gonadal cells

The above results indicated that the left-biased Pitx2 stimulated the expression of neurotransmitter receptors in the left developing female gonads. Previous studies suggested that neurotransmitters serotonin and dopamine could regulate the development of nonneuronal organs in chicken embryos (33). Thus, we speculate that Pitx2 could drive the left gonad development by enhancing serotonergic or dopaminergic signaling through the induction of neurotransmitter receptors (Htr1b or Drd4). In contrast, the right degenerating gonads are devoid of Pitx2 expression and thus lack the Htr1b or Drd4 receptor to activate the serotonergic or dopaminergic signaling pathways to sustain gonadal development. Thus, we tested this hypothesis by forcibly activating the residue serotonin and dopamine receptors in the right regressing gonad and examined the developmental dynamics afterward.

**Fig. 5.**
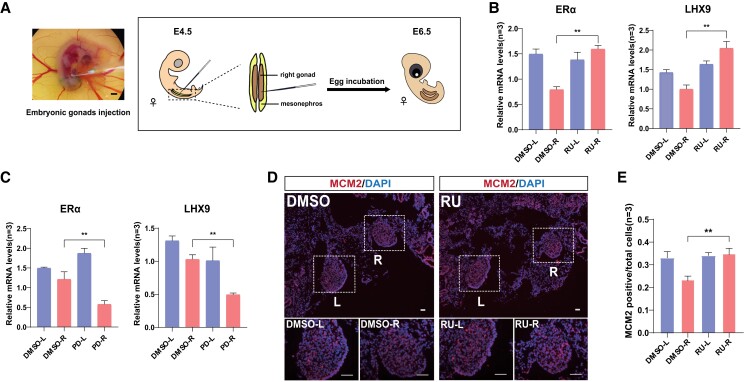
Activation of HTR1B, not DRD4, receptors in the female right gonads increased the expression of ovarian genes and gonadal cell proliferation. A) Schematic diagram showing the method for embryonic gonad injections. The agonists of HTR1B or DRD4 were injected into the right gonads at E4.5 and sample collection at E6.5. Scale bar: 1 mm. B) RT-qPCR showed the up-regulation of ERα and LHX9 upon HTR1B receptor activation by RU24969 (RU). C) The expression of ERα and LHX9 was down-regulated after DRD4 receptor activation by PD168077 (PD). D) Representative image showing MCM2 immunofluorescence staining in gonads after HTR1B receptor activation by RU24969 (RU). The white dashed box indicates the magnified area at the bottom showing single gonads. Scale bars: 100 μm. E) Quantification of MCM2 positive cells in gonads after HTR1B receptor activation by RU24969 (RU). Data are mean ± SEM; **P < 0.01 after the *t* test; *n* = 4 in each group.

At E4.5, the receptor agonists RU24969 (target HTR1B) and PD168077 (target DRD4) were injected near the right mesonephros of female chicken embryos. At the same time, DMSO was injected as a control. Gonads were collected at E6.5 and subjected to qPCR experiments and immunofluorescence staining to check the expression of gonadal marker genes and cell proliferation dynamics (Fig. 5A). ERα is the nuclear receptor that is essential for normal ovarian differentiation (34). LIM homeobox protein 9 (LHX9) is a transcription factor critical for gonad development (35). We found that the expression levels of ERα and LHX9 were significantly increased in the female right gonads upon the administration of HTR1B agonist RU24969 (Fig. 5B). However, the treatment of DRD4 agonist PD168077 reduced the expression of ERα and LHX9 (Fig. 5C). Hence, it seems that the activation of serotonin signaling, not dopamine signaling, could rescue the gonadal marker gene expression in the right regressing gonad.

Subsequently, we examined the cell proliferation dynamics in the RU24969-treated and control gonads by immunofluorescence staining for minichromosome maintenance protein 2 (MCM2) protein, which is a key component of the eukaryotic nuclear DNA prereplication complex and is involved in the entire cycle of cell proliferation (36). The results showed that the proportion of MCM2-positive cells in the right gonad was significantly increased and reached a comparable level with the left gonad (Fig. 5D and E). It indicates that forcibly activating serotonin signaling can promote gonadal cell proliferation and ovarian gene expression, thereby rescuing the development of the right regressing gonad to some extent.

**Fig. 6.**
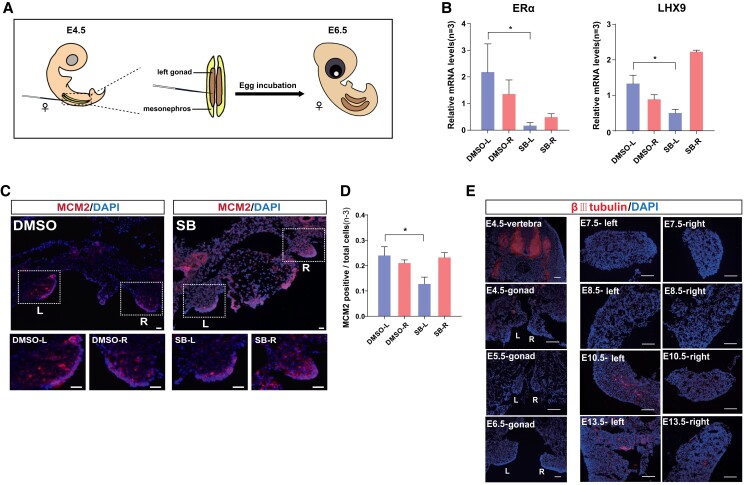
Blockage of HTR1B receptors in the female left gonads decreased the expression of ovarian genes and gonadal cell proliferation. A) Schematic diagram showing the method for embryonic gonad injections. The antagonists of HTR1B were injected into the left gonads at E4.5 and sample collection at E6.5. B) RT-qPCR showed the down-regulation of ERα and LHX9 upon HTR1B receptor inhibition by SB-224289A (SB). C) Representative image showing MCM2 immunofluorescence staining after HTR1B receptor inhibition by SB-224289A (SB). The white dashed box indicates the magnified area at the bottom showing single gonads. Scale bars: 100 μm. D) The proportion of proliferating cells in gonads after HTR1B receptor inhibition as quantified by MCM2 staining. E) Expression of βIII-tubulin in embryonic gonads during development. Scale bars: 500 μm. Data are mean ± SEM; *P < 0.05 after the *t* test; *n* = 4 in each group.

To further validate the major role of serotonin signaling in promoting gonad development, we conducted loss-of-function experiments in the left developing gonad (Fig. 6A). The commonly used HTR1B inhibitor (SB-224289A) (37) was injected into the left developing gonad to block the serotonin receptor. We found that the expression of ERα and LHX9 was significantly inhibited in the left gonads upon the administration of HTR1B inhibitors (Fig. 6B). Moreover, the number of proliferating cells in the left gonad decreased significantly upon treatment and was even slightly lower than the right gonad (Fig. 6C and D), indicating that blocking serotonin signaling with HTR1B inhibitors indeed suppressed ovarian gene expression and gonadal cell proliferation. Therefore, combining the loss-of-function experiments on the left developing gonads and the gain-of-function experiments on the right regressing gonads, we can confirm that HTR1B and serotonin signaling are necessary for gonadal development.

Current evidence suggests that neurotransmitters diffused in the blood or soft embryonic tissues can act as possible chemical signals to stimulate cell division and tissue development long before mature neurons appear in the embryo. Furthermore, the dimorphic expression of neurotransmitter receptors between the two female gonads may guide the formation of peripheral neurons. Thus, we continue to explore the distribution of neurons in the left and right gonads during development. We therefore tried to identify the nerve cells by immunofluorescence staining in the developing female gonads using βIII-tubulin, a marker of all types of mature neurons (38). The results showed no formation of mature neurons during early gonadal development (E4.5–E8.5), although abundant serotonin and dopamine receptors were already expressed at this stage. Positive signals for mature neurons appeared starting from E10.5, and the distribution in the left gonads was apparently more than that of the right (Fig. 6E). This suggests that the early emergence of neurotransmitter receptors may also be involved in stimulating the formation of later mature neurons in the gonads.

## Discussion

In avian embryos, the left/right asymmetry signals appear as early as during gastrulation. Unlike the motile cilia-induced leftward flow of fluid in the primitive node of mammals (39), birds employed asymmetric cell rearrangements to establish the leftward movement of cells around Hensen’s node, which produced a left-specific expression of Shh and FGF8 (40). The left-sided expression of Shh, in turn, would stimulate the left-sided expression of Nodal in a small domain just anterior to Hensen’s node. Nodal has a much broader expression domain occupying most of the left lateral plate mesoderm (LPM) (41, 42). Pitx2 was shown to be downstream of Nodal activity, but it persists much longer throughout organogenesis than Nodal (39). For instance, Nodal is not expressed in chicken gonads, but Pitx2 is specifically expressed in the left gonads of both sexes (5). Thus, we set out to identify the signaling module conferring left-sided instructions to Pitx2 in the developing gonads, particularly in females. We identified that the core sequence located within 1 kb upstream of the transcription initiation site of the Pitx2 gene had the strongest promoter activity. Among the predicted transcription factors that could bind to the core promoter region and also showed left-biased expression in the gonads, several transcription factors emerged to be involved in transcriptional activation, cell proliferation, and gonadal development. LHX1 encodes a LIM-like homeodomain transcription factor that is essential for the development of the mesonephric duct, mesonephros, metanephros, Müllerian duct, and embryonic gonads of mice. The LHX1 knockout mice lack the uterus and fallopian tubes, demonstrating the important role of LHX1 in the development of the female reproductive tract (28, 43). ATF1 is a member of the activating transcription factor family, which can bind to the upstream sequences of target genes to regulate gene expression. It is widely involved in physiological processes such as ion metabolism, cell proliferation, and apoptosis (44). STAT5 is a member of the STAT family (signal transducer and activator of transcription), which regulates cell cycle, cell survival, and angiogenesis and plays an important role in mammary gland development (45). The function of these left-biased transcription factors remains to be further investigated.

On the other hand, previous studies suggested that the persistent expression of Pitx2 in the female left gonad would promote germ cell proliferation and cortex formation in chicken embryos (16). However, the direct downstream targets of Pitx2 were not known. As a transcription factor, Pitx2 could bind to chromatin and directly regulate the transcription of target genes. We performed ChIP-seq experiments and identified the genome-wide binding sites and the potential target genes of Pitx2 in chicken genomes. After overlapping with the genes that were left-biased expressed in the gonads, we found that the genes encoding the neurotransmitter receptors were most enriched and the following functional validation experiments confirmed their involvement in gonadal development in chickens. It has been reported that neurotransmitters such as serotonin and dopamine can stimulate cell proliferation and gonadal development (46) and that neurotransmitter receptors were expressed in gonadal tissues in different species including mollusks (47–50) and teleosts (51, 52). Our data in chickens revealed that the two neurotransmitter receptor genes, HTR1B and DRD4, both have higher expression levels in the left gonad and show strong Pitx2 binding signals on their promoters. To explore whether these two neurotransmitter receptors could play a role in the development of the ovary, we activated HTR1B or DRD4 through the administration of receptor agonists into the right gonads at E4.5. We found that the activation of the serotonin pathway in the right regressing gonad could increase the expression of ovarian genes (ERα and LHX9) and the proliferation of gonadal cells. In contrast, blockage of the serotonin pathway in the left developing gonad leads to reduced ovarian gene expression and cell proliferation. Therefore, it seems that the serotonin pathway also supports gonad development in chickens, similar to that in fish and shrimp species (53–55). In other words, the left-specific Pitx2 directly stimulates HTR1B expression, which in turn sustains serotonin signaling to support the continued growth of the left gonad. In contrast, in the right gonad, the lack of Pitx2-induced HTR1B expression impaired serotonin signaling and could be responsible for gonad degeneration.

Neurotransmitters, such as serotonin, emerge in the embryo even before the neuron is formed. During embryogenesis, they may have broad functions other than neuromodulation, such as the stimulation of cell differentiation and neuronal cell growth (56). We confirmed that serotonin, a multifaceted molecule, may also act as a developmental morphogen in chicken gonads. In addition, the same molecule may be crucial for peripheral neuronal growth and the establishment of neuronal networks in various organs. In mice, neural crest-derived neurons emerge in the ovary only during the late stage of embryo development around E16.5 (57). This is consistent with current findings in chickens, where mature neurons first begin to appear in the female gonad from E10.5. The distribution of neurons in the left side was significantly greater than that in the right side, which is in line with the development of the left side and the degeneration of the right side. The left-specific expression of neurotransmitter receptors could promote the development of neurons in the chicken gonad, but it requires further study.

Taken together, we revealed a genetic program that directs the asymmetric development of female gonads in chickens. The left-biased Pitx2 directly activates HTR1B expression, which in turn sustains serotonin signaling in the left gonad. This leads to an increase in cell proliferation in the left gonad, resulting in the thickening of the cortex. However, mature neurons begin to form in the left gonad much later to support further ovary growth. These findings provide new insights into the intriguing and unique left-sided ovarian development in birds and offer an experimental basis for improving reproduction in poultry.

## Materials and methods

### Chicken embryonic fibroblast cell (DF-1) culture

DF-1 cells were cultured in the medium of 10% FBS (CELLiGENT, CG0430A) in RPMI 1640 (Gibco, C11875500BT) at 39°C in 5% CO_2_ with saturated humidity. The cells were subcultured when confluency reached ∼90%.

### Chicken embryo incubation and gonad collection

Fertilized eggs of White Leghorn chickens were incubated at 37°C and 60% humidity. The left and right gonads were detached from the mesonephros (primitive kidney) under a stereo microscope and cleaned in cold PBS. The isolated gonads were either snapped frozen in liquid nitrogen for RT-qPCR or fixed with 4% paraformaldehyde (PFA) for immunofluorescence staining. A small piece of embryonic heads or other tissues was collected and stored at −20°C separately for PCR sexing. All animal procedures were performed according to the protocols of Huazhong Agricultural University and the Institutional Animal Care and Use Committee.

### Genetic sexing

For the genetic sexing of embryos, a small piece of tissue was digested to extract DNA with a genomic DNA extraction kit (TIANGEN, DP304-02). Genetic sexing was carried out by a standard genotyping PCR protocol focused on the chicken CHD1 (Chromo-helicase-DNA-Binding) gene. The CHD1 primer is listed in Supplementary Table S1. These primers are targeting the introns of the CHD1 gene, which is located on the Z (CHD1Z, 482 bp) and W chromosomes (CHD1W, 326 bp) (58). PCR reactions were 95°C for 5 min followed by 30 cycles of 95°C for 30 s, 51°C for 30 s, 72°C for 30 s, and a final extension step of 72°C for 10 min. Amplicons were separated from one band (Z) in the case of male animals or two bands (Z + W) in the case of female animals on a 1% agarose gel.

### Construction and truncation of the dual-luciferase reporter vector from the PITX2 promoter

Using chicken blood DNA as a template, the 4,108 bp promoter fragment of PITX2 was generated by PCR using the primer PITX2-promoter-4k (listed in Supplementary Table S1); this was cloned into the pGL3-Basic vector. The 4,108 bp promoter fragment was truncated into 961 bp, 2,065 bp, and 3,446 bp fragments using different primers and cloned into the pGL3-Basic vector, respectively. PCR primer sequences are listed in Supplementary Table S1.

### Construction of the PITX2-FLAG fusion overexpression vector

Due to the lack of optimal antibodies against chicken PITX2, it is impossible to directly use gonadal cells for ChIP-seq experiments. Therefore, to mimic the expression state of PITX2 in the left gonad, we constructed a PITX2-FLAG fusion expression vector to transfect chicken embryonic fibroblast cells. Using chicken blood DNA as a template, the coding sequence (CDS) of PITX2 was generated by PCR using the primer PITX2-CDS (listed in Supplementary Table S1); this was cloned into the eGFP-pCW vector. In this fusion overexpression vector, the CDS of PITX2 is placed after the CMV promoter, and the added 3×FLAG-tagged protein fused in-frame with the CDS. A constitutive hPGK-eGFP is also included in the vector.

### ChIP-seq

The cells transfected with the PITX2-FLAG fusion expression vector or digested by the E4.5 female left gonads were collected for ChIP. Cells were cross-linked using 1% formaldehyde at room temperature for 10 min followed by quenching using 200 mm glycine for 10 min at room temperature. The parameters of sonication were 34% power and 20 s ON/30 s OFF cycles for 4 min. Chromatin was incubated at 4°C overnight with 0.5 mg/mL FLAG antibodies (Sigma, M8823) or 1 mg/mL ATF1 (Abcam, ab181569) or EBF1 antibodies (ABclonal, A12782). After incubation, beads were washed three times with lysis buffer (0.1% SDS, 1% Triton X-100, 1 mm EDTA, 50 mm HEPES, 150 mm NaCl) followed by two washes with high-salt wash buffer (0.1% SDS, 1% Triton X-100, 1 mm EDTA, 50 mm HEPES, 350 mm NaCl), then one wash with LiCl wash buffer (0.25 m LiCl, 0.5% NP40, 0.5% sodium deoxycholate, 1 mm EDTA, 10 mm Tris-HCl pH 8.0), and finally two washes with TE pH 8.0 (10 mm Tris-HCl, 1 mm EDTA). Beads were then resuspended in 90 μL of freshly prepared ChIP elution buffer (1% SDS, 0.1 m NaHCO_3_) and incubated at 65°C for 30 min with shaking (59). IP and input samples were treated with Proteinase K (Ambion, AM2546). DNA was then extracted with phenol/chloroform/isoamyl alcohol, precipitated with 3 m sodium, and resuspended in 10 mm Tris-HCl. ChIP-seq data have been generated on Illumina HiSeq X Sequencer.

### ChIP-seq analysis

For the data quality control of ChIP-seq data, Bowtie2 (version 2.3.5.1) was performed to gain read alignment by mapping reads against reference genome galGal6 (http://ftp.ensembl.org/pub/release-102/fasta/gallus_gallus/dna/). Peaks were called by MACS2 (version 2.1.1) with default parameters and were filtered with −log10(qValue) < 5 (60). The R programming language (version 4.0.3) and package ChIPseeker (version 1.26.0) were adopted to perform peak annotation (61). All alignment results were then converted to coverage bigwig files and normalized to the corresponding input using deepTools (version 3.0.2) (62). The bigwig formats can be visualized by software IGV. And the “mergePeaks” function was conducted to do peak overlap in order to determine the special and shared peak among these groups.

### Injection of the receptor activator of HTR1B and DRD4 and RT-qPCR analysis

Chicken embryos incubated for 4.5 days were used for injection experiments. A 1 cm^2^ window was made in the equatorial plane of the eggshell to expose the embryo. RU24969 hemisuccinate (the receptor agonist of HTR1B, Santa Cruz Biotechnology, SC-204165), PD168077 maleate (the receptor agonist of DRD4, Santa Cruz Biotechnology, SC-204896), and SB-224289A (the receptor inhibitor of HTR1B, MedChemExpress, HY-101105A) were dissolved in DMSO (2.5 μg/μL). The solutions were injected into the left or right gonadal ridge, located medial to the mesonephric kidney, with a glass capillary needle and PV830 pneumatic PicoPump machine. The injection volume was 1 μL per embryo, and the vehicle DMSO was used as control. Eggshells were sealed with medical tape, and injected embryos were carefully transferred to an incubator. Two days later, the E6.5 gonads were removed and cleaned rapidly, then snap frozen in liquid nitrogen and stored at −80°C individually for RNA extraction; others were fixed in 4% PFA overnight and incubated in 30% sucrose for 10 h at 4°C for immunofluorescence staining. A piece of head tissue was collected and stored at −20°C for determining the sex by PCR. At least three gonads were pooled together according to side and considered one biological replicate. Total RNA was extracted using a TRIzol reagent (Simgen, 5301100), and cDNA was prepared using a PrimeScript RT Reagent Kit with gDNA Eraser (Takara, RR047A). Analysis of mRNA expression was performed with an SYBR green RT-PCR kit (ABclonal, RM21203). All primers are listed in Supplementary Table S1.

### Immunofluorescence staining

The gonads were fixed with 4% PFA and dried on filter paper, then soaked in Tissue-Tek O.C.T. compound (Sakura Finetek Europe B.V.) before snap frozen in isopentane (2-methylbutane) cooled with liquid nitrogen. Sections were made at 5–10 µm thickness by Leica 1950 Cryostat. The sections were fixed again in 4% PFA, then permeabilized in 0.2% Triton X, blocking in 5% donkey serum. Antigen retrieval was performed in 10 mm pH 6.0 Trisodium Citrate (Sinopharm) at 75°C for 30 min as previously described (63). The sections were incubated with the HTR1B antibody (Bioss, bs-1125R, 1:500), DRD4 antibody (Boster, A00998-2, 1:500), MCM2 antibody (Abcam, ab4461, 1:200), and βIII-tubulin antibody (R&D Systems, MAB1195-SP, 1:500) overnight at 4°C and the corresponding secondary antibodies conjugated to Alexa Fluor 555 (Invitrogen, 1:500) for 2 h at room temperature. DAPI (50 μm) was used for nuclear staining (64). Three random sections were collected and five representative images were taken from each gonad, and MCM2+ cells were counted.

### Quantitative Real-Time PCR

Total RNA was isolated from transfected cells or collected gonad tissues by using the TRIzol reagent (Simgen, 5301100). cDNA was prepared using the PrimeScript RT Reagent Kit with gDNA Eraser (Takara, RR047A). Analysis of mRNA expression was performed with SYBR green fluorescent dye (ABclonal, RM21203). qPCR analysis was performed with a Bio-Rad CFX96/384 fluorescent quantitative PCR instrument. PCR primer sequences are listed in Supplementary Table S1.

### Statistical analysis

Student’s *t* test was used for comparison of the differences of gene expression levels observed by qPCR. All statistical tests were two-sided, and the significance was determined (*P < 0.05, **P < 0.01, ***P < 0.001).

## Supplementary material

Supplementary material is available at *PNAS Nexus* online.

## Supplementary Material

pgad202_Supplementary_Data

## Data Availability

The female gonad RNA-seq data were from PRJNA766745 (5) and PRJNA171809 (4). The Pitx2 ChIP-seq was deposited in the NCBI database: PRJNA860150.
