# Supplementary material for: A PITX2–HTR1B pathway regulates the asymmetric development of female gonads in chickens
Source: PNAS Nexus. 2023 Jun 19;2(6):pgad202. doi: 10.1093/pnasnexus/pgad202 (PMC10304771; doi:10.1093/pnasnexus/pgad202)
Supplement: pgad202_Supplementary_Data [file pgad202_supplementary_data.zip › PNASNEXUS-PNASNEXUS-2022-01017R-s01.docx]

**Supplementary Information**

**Additional file 1: Table S1.**

**Table S1.** List of primers used for this study.

| Primer name | Forward primer sequences | Reverse primer sequences |
| --- | --- | --- |
|  |  |  |
| PITX2-promoter-4k | GAACATTTCTCTATCGATAGGTACCCGCCTCGTTGAATTGCG | AGATCTCGAGCCCGGGCTAGCCGGCGAGAGGACGCCGGAGC |
| PITX2-promoter-1k | TGGAGTGGGGTTGCTGTCC | AGATCTCGAGCCCGGGCTAGCCGGCGAGAGGACGCCGGAGC |
| PITX2-promoter-2k | GTTACACAACGATCCCAGAG | AGATCTCGAGCCCGGGCTAGCCGGCGAGAGGACGCCGGAGC |
| PITX2-promoter-3k | TGCGCTACGAATCCCACG | AGATCTCGAGCCCGGGCTAGCCGGCGAGAGGACGCCGGAGC |
| PITX2-promoter-check | TAAGATGTCAGGCCAAGAGG | GAGAAGGAGCGCGTGTGC |
| CHD1 primer | TGCAGAAGCAATATTACAAGT | AATTCATTATCATCTGGTGG |
| PITX2-CDS | CTACCGGTCGCCACCATGGATG | GATAAGTGAGGATCCGGGGT |
| PITX2-qPCR | TGCTCCTCCTCACCTTCCTC | ATCCTCGCTCTTGCCCTG |
| DAZL-qPCR | GCCCAGTGTGGAAGTATCTCA | GAACATCCACATGTCCAGGAATG |
| Pou5f3-qPCR | CTCAATGAGGCAGAGAACACG | CAGACCCGGACAACGTCTTT |
| PIWIL1-qPCR | TCACCTGAGCAAAGACAAC | TCCCGTAAAGGACAGTAAG |
| DDX4-qPCR | TCCATCTTTGCATGTTATCAGTCAGG | AATCCCGCCCTGCTTGTATAACAG |
| SOX3-qPCR | GTGGGCCAGAGGATTGACAC | GGGACATGCTGTAGGTGGAG |
| TDRD9-qPCR | GGAAAAGGAGAAAACATCTGCATCT | TTCGTATGACGGCACTTCTGA |
| WNT11-qPCR | GCCTCCTATTTTCCCCTCGG | TTCTAGCTGCTTGCAGTGCT |
| ERα-qPCR | CAGATGGTCAGTGCCTTGCT | CGCCAGACTAAGCCGATCAT |
| LHX9-qPCR | ATACTTGTCCTTTCCGTCCC | TCATCTGTCCCCCTTTGG |
| HTR1B-qPCR | CACAGGCTGAACATTTGGAA | TGCCAGAGTCATTACACTGA |
| DRD4-qPCR | GCCCTGATGACCATGGATGT | GTTTGGGACGTTGTTGAGGC |
| GAPDH-qPCR | TGAAAGTCGGAGTCAACGGATT | ATAGTGATGGCGTGCCCATT |
| PITX2promoter-ChIPqPCR-region1 | CGCAGTTCAATGGCCTGATG | CATGCTGGAGGACATGCTCA |
| PITX2promoter-ChIPqPCR-region2 | CATGAGCATGTCCTCCAGCA | TGGCCAAGCTCGAGTTACAC |
| PITX2promoter-ChIPqPCR-region3 | GAAGGACCCGTTAAGCCTGG | AGGTGTCGGAGATAGTGTGC |
| CCNA1-ChIPqPCR | TCCAAGTTCTTGCCTGTTCCTT | TTCTGCGACTTGTCTGTGGAT |
| LEF1- ChIPqPCR | GCAGGTATCTGGGTCAAATGT | ACAGAGTGGGATGTGCATTG |

**Additional file 2: Table S2.**

**Table S2.** Information and mapping summary of ChIP-seq sequencing data.

| Data type | Factor | ID | Total reads | Total mapped rate |
| --- | --- | --- | --- | --- |
| ChIP-seq | PITX2 | DF1-FLAG-1 | 84,736,976 | 94.33% |
| ChIP-seq | PITX2 | DF1-FLAG-2 | 78,417,888 | 93.86% |
| ChIP-seq | None | Input | 88,785,252 | 92.64% |
