# Supplementary material for: A PITX2–HTR1B pathway regulates the asymmetric development of female gonads in chickens
Source: PNAS Nexus. 2023 Jun 19;2(6):pgad202. doi: 10.1093/pnasnexus/pgad202 (PMC10304771; doi:10.1093/pnasnexus/pgad202)
Supplement: pgad202_Supplementary_Data [file pgad202_supplementary_data.zip › PNASNEXUS-PNASNEXUS-2022-01017R-s02.docx]

**Supplementary Information**

**Figure S1.**

**
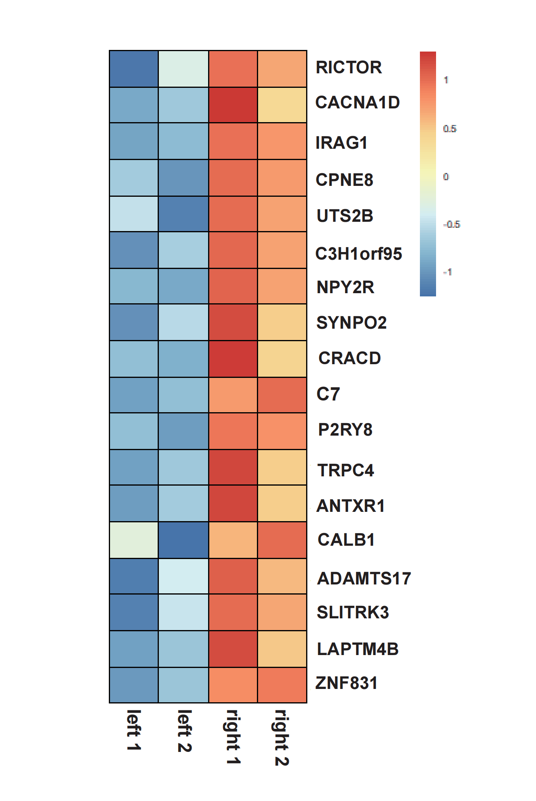
**

**Figure S1.** Display of up-regulated DEGs in female right of RNA-seq data.

**Figure S2.**


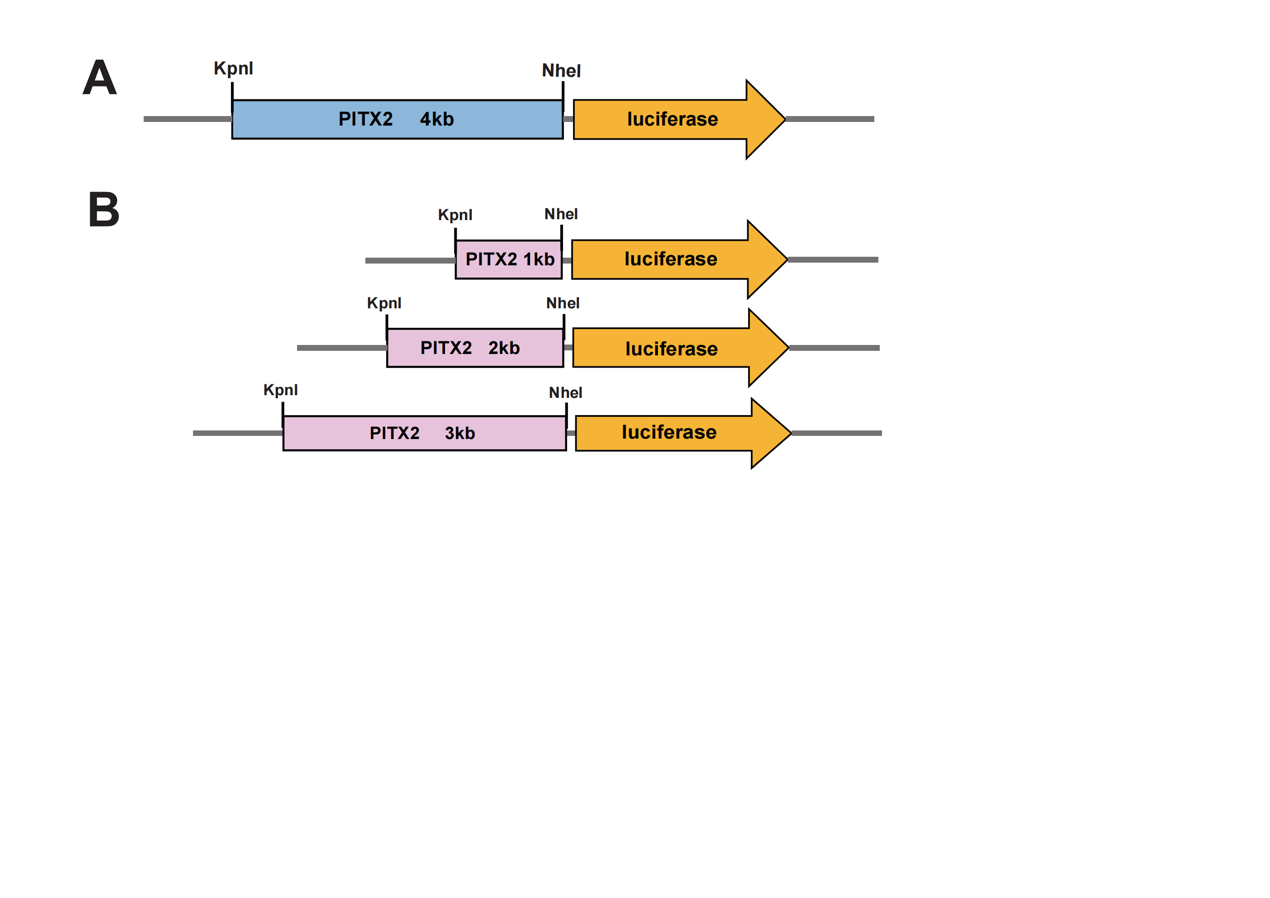


**Figure S2.** Schematic diagram of PITX2 promoter vector. (A) Schematic diagram of PITX2-promoter-4k vector. (B) Schematic diagram of pGL3-PITX2-promoter-1k, 2k, 3k vector.

**Figure S3.**


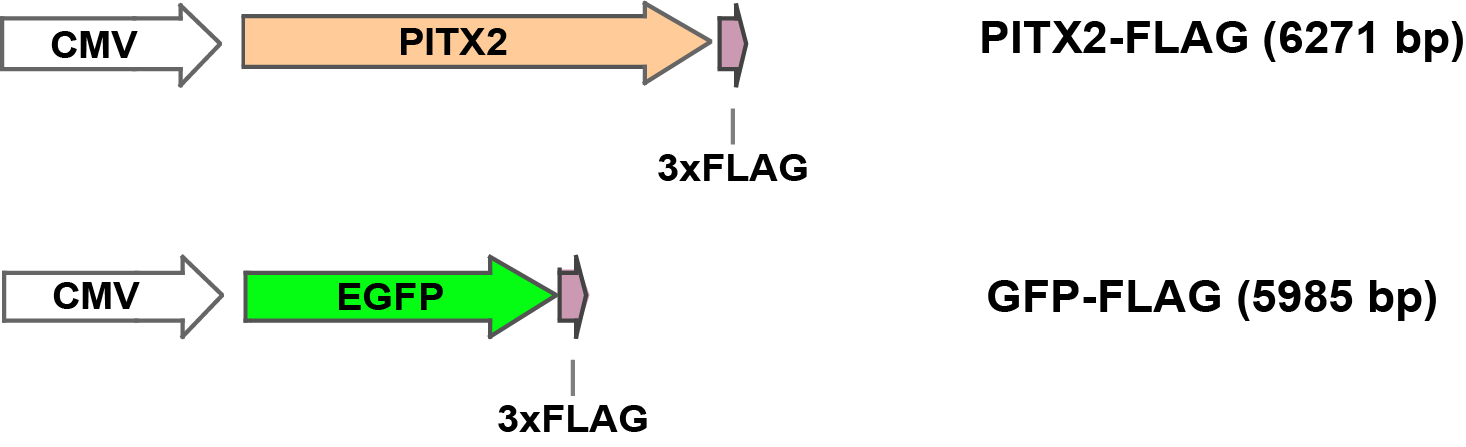


**Figure S3.** The plasmid structure for validation of ChIP experiments of the PITX2

and negative control. The PITX2+FLAG or GFP+FLAG was fused and expressed together.

**Figure S4.**


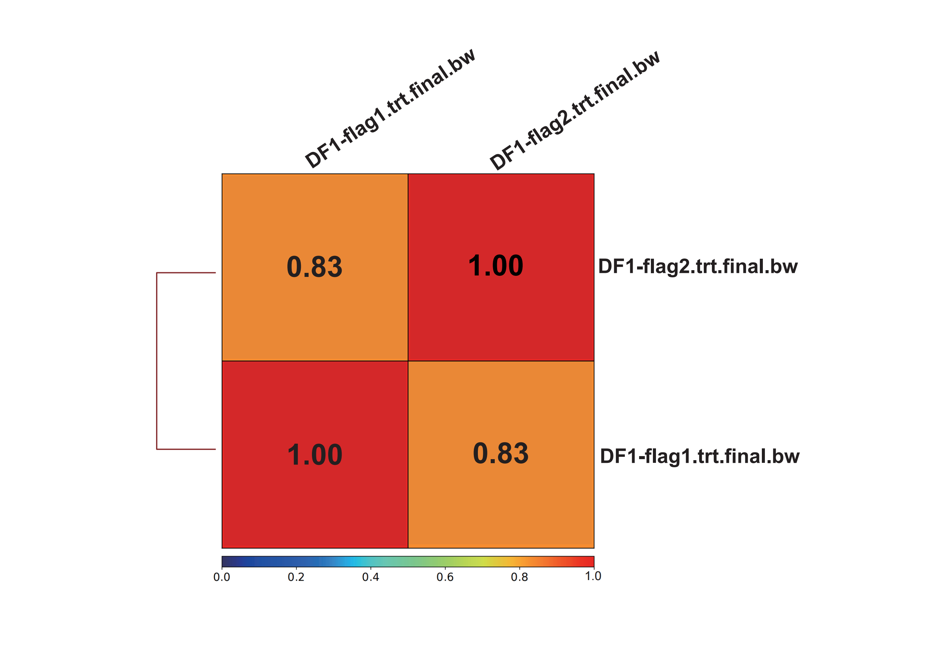


**Figure S4.** Correlation test between biological replicates of ChIP-seq data.

**Figure S5.**


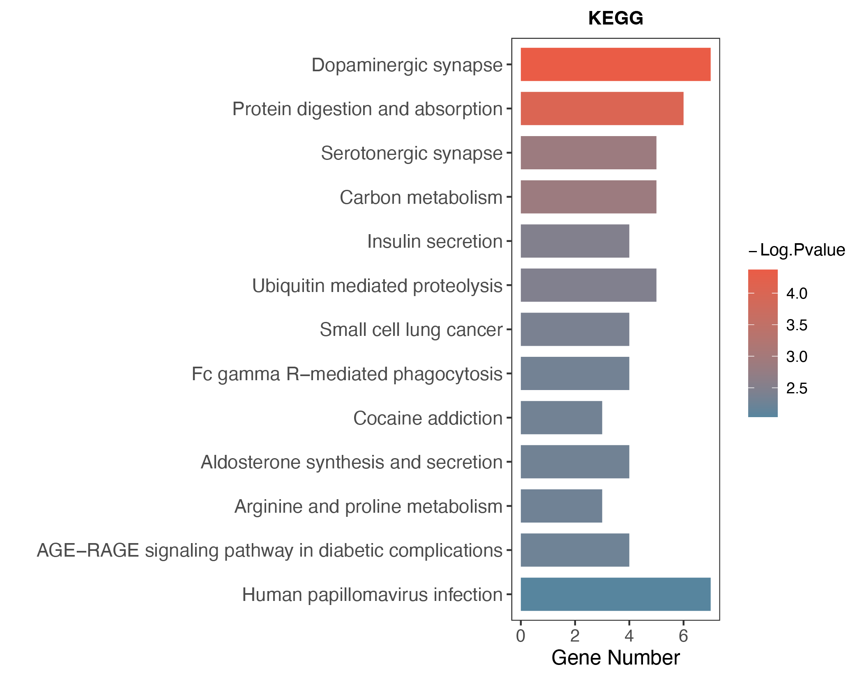


**Figure S5.** KEGG pathway analysis of the overlapping genes with left gonad specific expression in RNA-seq and genes with Pitx2 peaks located within 0-1 kb of TSS in ChIP-seq.
